# Physical activity counseling in primary health care in Brazil: a national study on prevalence and associated factors

**DOI:** 10.1186/1471-2458-13-794

**Published:** 2013-08-31

**Authors:** Alex Antonio Florindo, Gregore Iven Mielke, Grace Angélica de Oliveira Gomes, Luiz Roberto Ramos, Mário Maia Bracco, Diana C Parra, Eduardo J Simoes, Felipe Lobelo, Pedro Curi Hallal

**Affiliations:** 1 School of Arts, Sciences and Humanities, University of Sao Paulo, São Paulo, SP, Brazil; 2 Postgraduate Program in Epidemiology, Federal University of Pelotas, Pelotas, RS, Brazil; 3 Gerontology Department, Federal University of Sao Carlos, São Carlos, SP, Brazil; 4 Department of Preventive Medicine, Federal University of Sao Paulo, São Paulo, SP, Brazil; 5 Hospital Israelita Albert Einstein, São Paulo, SP, Brazil; 6 Prevention Research Center in St. Louis, George Warren Brown School of Social Work, Washington University in St. Louis, St. Louis, Missouri, USA; 7 School of Medicine, Department of Health Management and Informatics at University of Missouri, Columbia, Missouri, USA; 8 Division of Diabetes Translation, National Center for Chronic Disease Prevention and Health Promotion, Centers For Disease Control and Prevention, Atlanta, GA, Georgia; 9 School of Physical Education, Federal University of Pelotas, Pelotas, RS, Brazil

**Keywords:** Physical activity promotion, Physical activity counseling, Primary health care, Physicians, Nurses, Knowledge, Associated factors

## Abstract

**Background:**

The aim of this study was to determine the prevalence and correlates of physical activity counseling among physicians and nurses working in primary health care in Brazil.

**Methods:**

A phone survey was carried out in 2011 with professionals working in primary health care in Brazil. The target sample consisted of 1,600 randomly selected primary care units covering all regions of the country. We successfully interviewed 529 professionals within the sampled units; 182 physicians and 347 nurses. The overall response rate was 49.6%. Multivariable logistic regression was used to estimate correlates of counseling in the whole sample and separately for physicians and nurses.

**Results:**

The prevalence of regular physical activity counseling for at least six months was 68.9% (95% CI 64.9; 72.8) and was significantly higher among physicians compared to nurses (p < 0.05). Most professionals (93.2%) interviewed were unfamiliar with current physical activity recommendations for health. In the adjusted analysis, physical activity counseling was more frequent among those who report assessing patient’s physical activity (OR = 2.16; 95% CI 1.41; 3.29), those reporting that lack of time was not a barrier for counseling (OR = 0.62 95% CI 0.42-0.93), those who felt prepared to provide physical activity counseling (OR = 2.34; 95% CI 1.50-3.66), and those working at primary care units offering physical activity programs for patients (OR = 2.06; 95% CI 1.33-3.20). In the stratified analysis, only assessing patient’s physical activity was a significant correlate among physicians whereas assessing patient’s physical activity, feeling prepared to provide counseling and working in units with physical activity interventions were significant correlates among nurses.

**Conclusions:**

Physicians and nurses deemed physical activity counseling of great importance in primary health care in Brazil. However, in order to increase the quality of counseling and the number of professionals engaging in this activity, these health teams require greater knowledge about physical activity (global recommendations for health) as well as training on the application of instruments for assessing physical activity. Moreover, sufficient time must be allowed during consultations for the counseling process, and physical activity promotion programs should be implemented within the primary health care units.

## Background

Physical activity counseling for patients in primary care settings is an important component of health promotion [1]. It represents a brief intervention that can be used by health professionals with patients, ranging from basic conversation to more personalized discussion focusing on behavioral changes [2]. Randomized controlled trials have shown that adults of both sexes can increase their physical activity and fitness after receiving counseling in primary health care [3-9]. In particular, office-based screening of activity levels and physical activity counseling coupled with regular follow-ups and community support and referrals for physical activity, have been proven effective in increasing physical activity levels [10,11]. In addition, the cost of integrating physical activity counseling and referral schemes into primary health care teams has been found feasible and cost-effective [12,13].

Given the benefits of physical activity counseling for public health, an increasing number of surveys have been conducted, mostly in high-income countries, to evaluate its prevalence and correlates in primary health care settings [14-19]. These studies showed that the prevalence of counseling ranged from 19% to 76% [14,16-18]. Factors found to be consistently associated with physical activity counseling include feeling prepared to advise patients on physical activity, having sufficient time during consultation to provide counseling and the presence of physical inactivity related co-morbidities among patients [14-17]. In addition, a systematic review showed that the two main barriers to physical activity counseling perceived by primary care professionals were lack of time and limited knowledge [20].

There is currently a dearth of studies exploring the prevalence of physical activity counseling and its correlates in representative samples of primary care providers from low and middle income countries such as Brazil. This is particularly important because 80% of deaths from non-communicable diseases occurs in low and middle income countries [21]. The situation in Brazil is particularly interesting for studies of this type. The country has a universal health care system, on which 70% to 75% of the national population depends [22]. Therefore, the potential health and economic impact of universal, standardized physical activity counseling and referral systems is substantial. Hence, the aim of this study was to determine the prevalence and correlates of physical activity counseling among physicians and nurses working in primary health care settings in Brazil.

## Methods

This cross-sectional study was conducted as part of the second cycle of Project GUIA (Guide for Useful Interventions for Activity in Brazil and Latin America). The main goal of Project GUIA is to examine and promote evidence-based strategies to increase physical activity in Brazil and Latin America [23]. Further information can be found at http://www.projectguia.org/.

A telephone survey was carried out to assess knowledge, opinions and practices regarding promotion of physical activity among health care professionals working in primary health care units in Brazil. In the survey, interviewees included units’ coordinators, physicians, nurses and community health workers. The primary sampling units comprised the 42,486 primary health care facilities of the country, based on the Ministry of Health registry. Within these units, we systematically sampled 1,600 for the survey; these units covered all regions of the country. Within each sampled primary health care unit, we interviewed the coordinator and one health professional (physician, nurse or community health worker). In order to do so, we targeted the coordinator and the physician in unit 1, the coordinator and the nurse in unit 2 and the coordinator and the community health worker in unit 3, for example, so that the total target sample included 1,600 coordinators, 534 physicians, 533 nurses and 533 community health workers. The last sampling unit was the specific health professional to be interviewed, because some units would have more than one physician, nurse or community health worker. In such cases, we asked the coordinator to list all physicians (nurses or community health workers) working in that unit and sampled one of them using a table with random numbers.

The present analysis was based on data from physicians and nurses only, for a total of 529 professionals (182 physicians and 347 nurses) interviewed. The overall response rate was 49.6% across all professionals eligible (34.1% for physicians and 65.1% for nurses). We opted not to use data from community health workers because the nature of their work is different from the other professionals, particularly due to the fact that instead of staying at the unit, community health workers spend most of their days visiting patients in their homes and prompting them to visit the unit whenever needed.

Data collection was carried out between January and July 2011. The questionnaires were administered over the telephone by a team of six previously trained interviewers. Calls were made to the coordinator of each unit. If he/she was unavailable, we tried to schedule the call for another hour or day. After interviewing the coordinator, we then sampled one health professional for his/her interview. Interviews lasted, on average, 40 minutes. The Ministry of Health sent a letter to the sampled units in order to encourage their participation in the survey.

### Questionnaires

Physical activity counseling was assessed by one question containing six response categories: “(1) I do not recommend physical activity and do not intend to start doing so; (2) I do not recommend physical activity, but I am thinking about starting to do so; (3) I sometimes recommend physical activity but do not do so on a regular basis; (4) I regularly recommend physical activity, but have started doing so only recently; (5) I’ve been regularly recommending physical activity for over 6 months; (6) I used to recommend physical activity, but no longer do so”. The questions used to assess professionals’ opinions regarding counseling, instruments used for patient’s physical activity assessment and knowledge about physical activity are presented in (Table 1).

**Table 1:** Questionnaire used for data collection

***Questions about physical activity counseling***	**Possible answers**
***Relevance***	
It is important that physical activity programs for the community are offered by the health system.	(a) Strongly disagree (b) Disagree (c) Agree (d) Strongly agree
Viability of offering physical activity programs at primary health care units.	(a) Yes (b) No
Priority in offering physical activity programs in health units.	(a) Yes (b) No
Who is the main health professional responsible for promoting physical activity in the unit?	(a) Physician (b) Nurse (c) Physical education professional (d) Nutritionist (e) Physiotherapist (f) Others
What difficulties do you face with regard to physical activity counseling?	(a) Lack of knowledge (b) Lack of time (c) Lack of places for physical activity practice nearby the unit (d) Others
***Physical activity assessment***	
Do you usually ask about the physical activity level of your patients?	(a) Yes (b) No
If so, what method do you employ?	(a) General questions about engagement in physical activity (b) Specific questions about sedentary time (c) Questions about duration, type and intensity of activities (d) Standardized questionnaires (e) Other method
***Knowledge about physical activity guidelines***	
How do you rate your knowledge about the current recommendations on physical activity for health?	(a) Know enough (b) Would like to learn more (c) Have insufficient knowledge
What is the minimum number of days per week that individuals should perform of physical activity of moderate intensity in order to achieve health benefits?	__ days per week
On these days, what is the minimum time recommended to obtain health benefits?	__ __ __ minutes
How should moderate-intensity physical activity be performed to have a positive impact on health?	(a) One bout per day (b) Can be done in 2–3 bouts per day
What is the minimum number of days per week that individuals should perform of physical activity of vigorous intensity in order to achieve health benefits?	__ days per week
On these days, what is the minimum time recommended to achieve health benefits?	__ __ __ minutes
How should vigorous-intensity physical activity be performed to have a positive impact on health?	(a) One bout per day (b) Can be done in 2–3 bouts per day
People can combine moderate-intensity activities (e.g. walking) with vigorous-intensity activities (e.g. running) for meeting physical activity guidelines?	(a) Strongly disagree (b) Disagree (c) Agree (d) Strongly agree

Data concerning the availability of free physical activity classes or physical activity programs for patients in the primary health care units’ catching area was obtained by administering a separate questionnaire to unit coordinators. Social and demographic characteristics of respondents were assessed including age, skin color, sex, educational level and variables related to professional practice at the health unit (number of hours per day spent on the unit, number of patients seen per week, and time working at the specific unit).

### Data analysis

We initially describe the counseling practices of health professionals using the six categories of response. For the analysis of correlates, providers who reported having regularly recommended physical activity for over six months were considered to provide counseling to patients. The prevalence of physical activity counseling was compared across categories of the independent variables using chi-square tests. Correlates found to be significantly associated (p < 0.05) with physical activity counseling in the unadjusted analyses were included in logistic regression models. Adjustment variables used were sex, age, duration of employment health professional (Physicians or Nurses) and number of patients seen per week. We then repeated the analyses separately for physicians and nurses. All analyses were run using SPSS 15.0.

### Human subjects

The project was approved by the Research Ethics Committee of the Physical Education School of the Federal University of Pelotas.

## Results

Nursing professionals (N = 347) were predominantly women (84.7%), had white skin color (62.0%) and a mean age of 32.6 years (SD 8.5). Most of them (88.5%) held no degree in public health. In terms of work experience, 93.3% graduated less than 10 years ago, and 81.8% worked for fewer than five years in primary health care units. Most nurses (77.2%) had a working week of 40 hours or longer. Physicians (N = 182) were predominantly men (56.6%), white (67.6%) and with a mean age of 40.5 years (SD 12.8). Again, most (90.7%) had no degree in public health. In terms of work experience, 65.2% graduated less than 10 years ago, and 81.7% worked for fewer than five years in primary health care units. Just over half (54.4%) had a working week of 40 hours or longer (Table 2).

**Table 2:** Socio-demographic characteristics of the physicians and nurses interviewed

**Variables**	**All**		**Physicians**		**Nurses**	
	**%**	**N**	**%**	**N**	**%**	**N**
**Gender**						
Male	29.5	156	56.6	103	15.3	53
Female	70.5	373	43.4	79	84.7	294
**Age (years)**						
≤30	44.1	233	26.9	49	53.2	184
31 to 40	31.1	164	31.9	58	30.6	106
>40	24.8	131	41.2	75	16.2	56
**Skin color**						
White	63.9	338	67.6	123	62.0	215
Others	36.1	191	32.4	59	38.0	132
**Specialization degree in Public or Family Health**						
Yes	10.8	57	9.3	17	11.5	40
No	89.2	447	90.7	165	88.5	307
**Time since the last academic degree (years)**						
<5	68.3	359	50.8	92	77.4	267
5 to 10	15.4	81	14.4	26	15.9	55
>10	16.3	86	34.8	63	6.7	23
**Working hours per week**						
<40	30.6	161	45.6	82	22.8	79
≥40	69.4	365	54.4	98	77.2	267
**Working time at the health care unit (years)**						
≤1	37.9	198	49.7	90	31.7	108
2 to 5	43.9	229	32.0	58	50.1	171
>5	18.2	95	18.3	33	18.2	62
**Patients attending per week**						
≤80	54.0	271	29.2	52	67.6	219
80 to 160	33.7	169	50.0	89	24.7	80
>160	12.3	62	20.8	37	7.7	25
Total	100	529	100	182	100	347

Very few professionals (0.3% of the nurses and none physicians) reported not recommending physical activity at all and having no plan to start doing so. Exactly the same proportions were true for the category “I do not recommend physical activity, but plan to start doing so”. The proportion of health professionals reporting to recommend physical activity sometimes, but not on a regular basis, was 21.8% in the whole sample, 12.2% among physicians and 26.9% among nurses. The proportions reporting to have started to recommend physical activity recently were 8.5% in the whole sample, 6.1% among physicians and 9.8% among nurses. The prevalence of regular physical activity counseling for at least six months were 68.9% (95%CI 64.9; 72.8) in the entire sample, 81.2% among physicians and 62.4% among nurses (Table 3). Only 0.4% of the professionals reported recommending physical activity regularly in the past, but not currently.

**Table 3:** Prevalence, knowledge and opinions of health professionals with regards to physical activity counseling, Brazil, 2011

**Variables**	General*	**Physicians**	**Nurses**
	**%**	**%**	**%**
***Physical activity counseling***			
Yes	68.9	81.2	61.4
Performs physical activity assessment*			
Yes	64.0	73.6	58.8
***Considers it feasible that physical activity programs are offered in your health care unit***			
Yes	86.0	83.4	87.3
***Considers it a priority that physical activity programs are offered in your health care unit***			
Yes	75.7	76.1	75.5
***Thinks the lack of knowledge is a barrier to physical activity counseling***			
Yes	41.3	37.0	43.5
***Thinks the lack of time is a barrier to physical activity counseling*** *			
Yes	64.2	59.7	66.6
***Thinks the lack of places for physical activity practice is a barrier to physical activity counseling***			
Yes	77.8	80.0	76.7
***Feels prepared to advise patients on physical activity*** *			
Well prepared	54.1	72.5	44.4
***Importance of physical activity programs in primary health care settings***			
Agree	94.7	95.0	94.5
***Who should be the main professional responsible for physical activity promotion in primary health care settings?***			
Physical Education professional	58.5	44.8	65.7
***Self-rated knowledge on the recommendations of physical activity for health***			
Do not know enough	92.6	84.8	96.5
***Knowledge on the recommendations for moderate-intensity physical activity***			
Responded incorrectly (duration or frequency)	93.2	93.4	93.1
***Knowledge on the recommendations of vigorous-intensity physical activity***			
Responded incorrectly (duration or frequency)	98.9	97.8	99.4
***Opinion on the possibility of combining moderate and vigorous-intensity physical activity for achieving health guidelines***			
Agree	73.3	71.2	74.4
The health care unit has physical activity programs for patients*			
Yes	40.5	46.3	37.4

*Significant associations (p < 0.05) with regular counseling for physical activity.

The majority of professionals reported assessing the physical activity of patients and considered the promotion of physical activity at the health units to be both feasible and a priority. Regarding the methods used for assessing physical activity of patients, most (77.4%) reported using general questions, while only 10.7% employed standardized questionnaires. Lack of time and of facilities for patient’s to engage in physical activity were the main barriers reported for providing physical activity counseling. Despite feeling prepared to provide counseling on physical activity, few primary care professionals answered correctly questions on physical activity recommendations for health (Table 3).

All the variables significantly associated with physical activity counseling in the bivariate analyses (Table 3) remained significant in the logistic regression models (Table 4), including conducting physical activity assessment on patients, feeling prepared to advise about physical activity, reporting lack of time as a barrier, and working on units offering physical activity programs for patients.

**Table 4:** Variables associated with regular counseling for physical activity among health professionals, Brazil, 2011

**Variables**	**All**		**Physicians**		**Nurses**	
	OR (CI95%)*	OR (CI95%)**	OR (CI95%)*	OR (CI95%)***	OR (CI95%)*	OR (CI95%)***
Performs physical activity assessment*						
No	1	1	1	1	1	1
Yes	2.35 (1.61-3.44)	2.15 (1.41-3.29)	1.68 (0.76-3.73)	2.57 (1.05-6.26)	2.32 (1.48-3.62)	2.14 (1.31-3.49)
Thinks the lack of time is a barrier for physical activity counseling*						
No	1	1	1	1	1	1
Yes	0.62 (0.42-0.93)	0.62 (0.40-0.98)	0.66 (0.30-1.44)	0.71 (0.30-1.66)	0.65 (0.41-1.04)	0.60 (0.35-1.02)
Feels prepared to advise patients on physical activity*						
Unprepared	1	1	1	1	1	1
Well prepared	2.00 (1.38-2.91)	2.36 (1.51-3.70)	1.11 (0.49-2.54)	1.68 (0.67-4.16)	1.89 (1.20-2.96)	2.62 (1.54-4.44)
The health care unit has physical activity program for patients*						
No	1	1	1	1	1	1
Yes	1.94 (1.31-2.89)	2.08 (1.34-3.23)	1.31 (0.61-2.80)	1.53 (0.68-3.44)	2.11 (1.31-3.40)	2.46 (1.45-4.20)

*Unadjusted analysis; **Adjusted for sex, age, health professional (Physicians or Nurses), working time at the unit health care and numbers of patients attended weekly; ***Adjusted for sex, age, working time at the unit health care and numbers of patients attended weekly.

In Table 4, we present the statistically significant results of physical activity counseling associations of with variables selected in Table 3 for three groups of respondents: the combined responses of physicians and nurses (health professionals) and stratified for professional group. Physical activity assessment was a significant correlate among health professionals, physicians and nurses. Feeling prepared to advise about physical activity, and working in health care units offering physical activity programs for patients were significant predictors of physical activity counseling among health professionals and nurses. Stating that lack of time was a barrier for physical activity counseling decreased the odds of physical activity counseling among health professionals and nurses.

**Figure 1:**
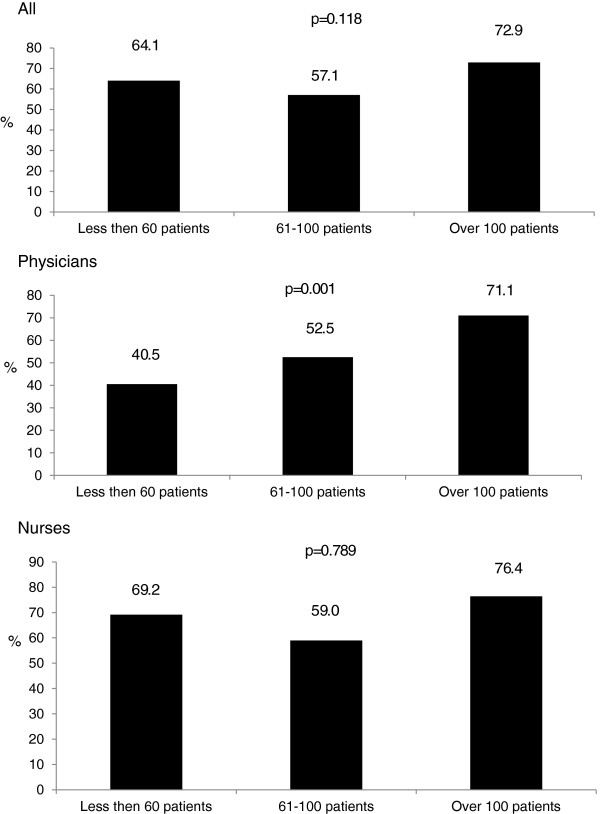
Prevalence of the report of lack of time as a barrier for physical activity counseling according to the number of patients seen by health professionals on a weekly basis.

Finally, the higher the number of patients attended weekly, the higher the prevalence of reported lack of time as a barrier for physical activity counseling (Figure 1).

## Discussion

Health professionals who reported assessing patients’ physical activity levels, who did not feel that lack of time was a barrier for them to counsel, those who felt well prepared to advise patients about physical activity, and who worked in health units offering physical activity programs for patients, had a greater likelihood of providing physical activity counseling in primary health care settings in Brazil. Assessing physical activity of patients was a significant correlate among health professionals, physicians and nurses. In addition, overall rates of physical activity counseling were high, with 8 out of 10 physicians and 6 out of 10 nurses reporting regular physical activity counseling to patients. However, in spite of these high counseling rates, their knowledge about physical activity recommendations was found to be relatively low.

In our sample, physicians were more likely than nurses to advice patients about physical activity, differently from a survey involving primary health care physicians and nurses in Scotland [14]. The Scottish study, although reporting a similar overall prevalence of physical activity counseling (76%), found rates of 62% among physicians and 90% among nurses. Another study with a sample of physicians in Estonia found that 94% reported advising patients with chronic diseases about physical activity [24]. Another survey among North-American nurses [25] found a prevalence of regular physical activity counseling of 57%. The prevalence of physical activity counseling reported here, in Scotland and Estonia were greater than the rates reported in both the U.S. and South Korea [17].

Our results are extremely important for the Brazilian scenario. The universal public health system in Brazil is the source of health care for 70 to 75% of the population. Therefore, physical activity promotion in this setting is highly viable, since health professionals can act as advocates for the promotion of healthy behaviors. Although there are no written specific Brazilian physical activity guidelines focused on health promotion, physical activity current recommendations have been met in other guidelines of chronic diseases published by the Ministry of Health. This situation is different from other places. For example, in the United States, physical activity counseling by primary health care providers constitutes a national public health objective [10].

Mirroring the results of the present study, a South Korean study showed that physicians who felt better prepared were more likely to promote physical activity [17]. However, this finding should be interpreted with caution, because out of the health professionals who felt prepared to provide physical activity counseling in the present study, most failed to correctly identify current guidelines for physical activity recommendations. In addition, no association was found between feeling prepared to counsel on physical activity and correctly identifying recommendations for physical activity. Similarly, the Scottish study also showed that few professionals had adequate knowledge to counsel about physical activity with regard to intensity and duration of exercise [14]. This highlights an urgent need for training of primary care professionals around the world in an effort to improve their level of knowledge regarding physical activity guidelines.

In this study, routine assessment of patient’s physical activity was associated with a greater likelihood of providing physical activity counseling. Although we found no other studies reporting this association, it is clear that such assessment reflects a more organized and concerted effort towards regular physical activity counseling. Nevertheless, a large proportion of primary health care professionals have not been adequately trained to assess physical activity. A survey conducted in the U.S. showed that most physicians used general questions to assess physical activity, while only 7% applied standardized questionnaires [18]. A similar result was found in the present study, where only 10.7% of health professionals reported using standardized instruments. A qualitative study carried out among primary care professionals in Australia showed that physical activity assessment is only routinely conducted in the presence of disease or risk factors [26]. This is important because it suggests that the implementation of a comprehensive program of physical activity counseling, including a standard initial assessment for all users is likely to have a significant impact on the promotion of physical activity. For example, physical activity “vital signs” in electronic medical records have been validated in clinical settings [27].

Health care professionals interviewed in the present study who did not report lack of time as a barrier were more likely to regularly counsel patients about physical activity. Physicians and nurses from both Scotland and South Africa cited lack of time as a barrier to providing advice on physical activity [14,15], and similar results were obtained in a recent systematic review of perceptions of physical activity counseling in clinical settings [20]. One of the most notable findings of the present study was that, as the weekly patient case load rose, the prevalence of reporting lack of time for regular physical activity counseling as a barrier also increased, particularly among physicians. This finding suggests that seeing an excessive number of patients per week may jeopardize health professionals’ work in Brazil’s primary health care system.

A greater likelihood of providing physical activity counseling was observed among health professionals from units that offered physical activity classes or physical activity programs for users. Therefore, it is important to discuss the implementation of an integrated network for the promotion of physical activity in the public health system. A study in elderly individuals from a low socioeconomic community in São Paulo showed that their perception of having primary health care units in their immediate neighborhood was associated with a greater likelihood of participation in leisure-time physical activity [28]. A qualitative study on factors influencing physicians and nurses in their management of risk factors for chronic disease, identified the importance of having infrastructure available to engage in physical activity, given that the advice is generally accompanied by encouragement to join structured exercise programs at gyms, clubs and health units [26]. Therefore, actions promoting physical activity interlinked with primary health care are essential. The ‘Academia da Cidade’ program implemented in the cities of Recife and Aracaju exemplifies this integration whereby centers and facilities to engage in physical activity had direct links with primary health care units [29,30].

A potential limitation of this study may have been overestimation of reported physical activity counseling by Brazilian health professionals, given that levels were higher than those obtained for primary care physicians in both the U.S. (30-50%) [19] and South Korea (51.1%)[17]. However, prevalence levels were similar to those of a cohort of physicians and nurses in Scotland (76% prevalence)[14] and the family physicians in Estonia (94% prevalence) [24]. In a recently published study among patients attending primary health care units of Southern and Northeastern Brazil, 28.9% of adults and 38.9% of older adults reported having received counseling for physical activity [31]. Another survey among German older adults showed that 32.8% of patients interviewed had been given advice on physical activity by physician and nurse practitioners [32]. These data indicate that the prevalence of physical activity counseling reported by health professionals differs from those reported by patients. However, it is important to take into account that patients’ self-reports can be affected by their understanding on the meaning of physical activity advice given by health professionals, leading to potential underestimates [33]. A population-based epidemiological survey of an adult urban population in Southern Brazil showed that the prevalence of physical activity counseling was 56.2%, but counseling was mostly done by physicians (92.5%) [34]. It is important to highlight that physical activity promotion is a governmental priority in Brazil. Since the implementation of the Health Promotion Policy in 2006, cities have been funded to develop physical activity interventions.

Another limitation of our study is the low response rate for physicians, which does reduce our statistical power for the analyses stratified by occupation. The study with South Korean physicians had a similar response rate (38.0%) [17] but other surveys on physical activity counseling among physicians from the U. S. and Scotland had higher response rates [14,18]. Notably however, some of the characteristics of the physicians interviewed in the present study were similar to those of physicians working within the primary health care in Brazil. A survey carried out on physicians working under the Family Health Strategy showed that the majority of health professionals were 40 years of age or younger (60%), predominantly male (56%), had not undertaken a specialized training course after graduating (70%) and had graduated within the last 15 years (63%) [35]. A final limitation is the definition of regular counseling for the practice of physical activity. While physicians and nurses may have talked about physical activity with patients, some of these activities might not be structured, planned and repeated [2].

## Conclusions

In conclusion, the results of this study showed that physicians and nurses deemed physical activity promotion and counseling of great importance in primary health care in Brazil. However, these health teams would benefit from continued education programs to improve their knowledge regarding physical activity since few demonstrated familiarity with current physical activity recommendations or applied standardized instruments for assessing physical activity of patients. Standardized education strategies are therefore needed [22], similarly to what happens in other developed countries that have universal health care system [11,13,36]. Finally, because physical activity levels are not increasing in Brazil [37], interventions are urgently needed to promote more physical activity counseling in primary health care. These interventions must be linked to the recently launched *‘Academia da Saúde’* program [38], a nationwide policy that will fund over 4,000 cities to build capacity for physical activity promotion by the year 2015.

## Competing interests

The authors declare that they have no competing interests.

## Authors’ contributions

AAF, GIM, GAOG, LRR and PCH contributed to the idea this study. AAF, GIM and PCH contributed for data analysis, interpretation and drafted the manuscript. GAOG, LRR, MMB, DCP, EJS and FL contributed to drafting and critically revising the manuscript. All authors have read and approved the final manuscript.

## Pre-publication history

The pre-publication history for this paper can be accessed here:

http://www.biomedcentral.com/1471-2458/13/794/prepub
